# Association of depressive symptoms and sleep disturbances with survival among US adult cancer survivors

**DOI:** 10.1186/s12916-024-03451-7

**Published:** 2024-06-05

**Authors:** Ailin Lan, Han Li, Meiying Shen, Daxue Li, Dan Shu, Yang Liu, Haozheng Tang, Kang Li, Yang Peng, Shengchun Liu

**Affiliations:** 1 Department of Breast and Thyroid Surgery, the First Affiliated Hospital of Chongqing Medical University, Chongqing, China; 2 Department of Critical Care Medicine, the First Affiliated Hospital of Chongqing Medical University, Chongqing, China; 3 Department of Breast and Thyroid Surgery, the Women and Children’s Hospital of Chongqing Medical University, Chongqing, China

**Keywords:** Depression, Sleep, Cancer survivors, Mortality, NHANES

## Abstract

**Background:**

Depression and sleep disturbances are associated with increased risks of various diseases and mortality, but their impacts on mortality in cancer survivors remain unclear. The objective of this study was to characterize the independent and joint associations of depressive symptoms and sleep disturbances with mortality outcomes in cancer survivors.

**Methods:**

This population-based prospective cohort study included cancer survivors aged ≥ 20 years (*n* = 2947; weighted population, 21,003,811) from the National Health and Nutrition Examination Survey (NHANES) 2007–2018 cycles. Depressive symptoms and sleep disturbances were self-reported. Depressive symptoms were assessed using the Patient Health Questionnaire 9 (PHQ-9). Death outcomes were determined by correlation with National Death Index records through December 31, 2019. Primary outcomes included all-cause, cancer-specific, and noncancer mortality.

**Results:**

During the median follow-up of 69 months (interquartile range, 37–109 months), 686 deaths occurred: 240 participants died from cancer, 146 from heart disease, and 300 from other causes. Separate analyses revealed that compared with a PHQ-9 score (0–4), a PHQ-9 score (5–9) was associated with a greater risk of all-cause mortality (hazard ratio [HR], 1.28; 95% CI, 1.03–1.59), and a PHQ-9 score (≥ 10) was associated with greater risk of all-cause mortality (HR, 1.37; 95% CI, 1.04–1.80) and noncancer mortality (HR, 1.45; 95% CI, 1.01–2.10). Single sleep disturbances were not associated with mortality risk. In joint analyses, the combination of a PHQ-9 score ≥ 5 and no sleep disturbances, but not sleep disturbances, was associated with increased risks of all-cause mortality, cancer-specific mortality, and noncancer mortality. Specifically, compared with individuals with a PHQ-9 score of 0–4 and no sleep disturbances, HRs for all-cause mortality and noncancer mortality in individuals with a PHQ-9 score of 5–9 and no sleep disturbances were 1.72 (1.21–2.44) and 1.69 (1.10–2.61), respectively, and 2.61 (1.43–4.78) and 2.77 (1.27–6.07), respectively, in individuals with a PHQ-9 score ≥ 10 and no sleep disturbances; HRs for cancer-specific mortality in individuals with a PHQ-9 score ≥ 5 and no sleep disturbances were 1.95 (1.16–3.27).

**Conclusions:**

Depressive symptoms were linked to a high risk of mortality in cancer survivors. The combination of a PHQ-9 score (≥ 5) and an absence of self-perceived sleep disturbances was associated with greater all-cause mortality, cancer-specific mortality, and noncancer mortality risks, particularly in individuals with a PHQ-9 score (≥ 10).

**Supplementary Information:**

The online version contains supplementary material available at 10.1186/s12916-024-03451-7.

## Background

The number of cancer survivors worldwide is growing rapidly, with 1.95 million new cancer cases and 600,000 cancer-related deaths expected in the USA in 2023 [1]. Cancer survivors are at risk of physical and mental illnesses in the years following the completion of treatment. Survivors have higher rates of sleep disturbances and depression than the general population, with reported prevalence rates of approximately 50% and 10%, respectively, and the rate of sleep disturbances among cancer survivors is approximately three times that of the general population [2, 3].

Depression is characterized by affective apathy, mood dysregulation, and diminished motivation [4]. In cancer survivors, depression can lead to a variety of negative outcomes, including longer hospitalization, poor adherence to anticancer treatment, a decline in quality of life, and an increased risk of suicide [5, 6]. In addition, depression itself may alter immunological, endocrine, and neurological functions, which might increase vulnerability to illness and result in death [7]. The American Society of Clinical Oncology (ASCO) provides practical recommendations for screening, assessments, and interventions for cancer survivors experiencing depression based on the Pan-Canadian guidelines [8].

Sleep disturbances, which include difficulties with various sleep patterns and aspects, such as insomnia, narcolepsy, sleep breathing disorders, and restless legs syndrome [9], are associated with quality of life in cancer survivors. Cancer survivors may be at increased risk of sleep disturbances during cancer diagnosis, treatment, and continued survival. Cancer therapies such as chemotherapy and radiation can aggravate or even cause sleep disturbances [10]. Sleep management in cancer survivors is an important issue; for this reason, the National Comprehensive Cancer Network (NCCN) modified its survivorship care recommendations in 2014 and updated them in version 1.2023, which now recommends routine sleep disturbance screening [11]. Various preclinical studies are also actively exploring the relationship between sleep and cancer, and interestingly, recent studies have shown that cancer cells spread aggressively during sleep, which has been linked to sleep-accelerated cancer development [12, 13]. This finding has generated a great deal of interest in the issue of sleep in cancer patients.

Furthermore, the relationship between depression and sleep disturbances is complex. In patients with depression, sleep complaints are prevalent in approximately 90% of patients [14]. Sleep disturbances are the most prominent symptom in patients with depression and were previously thought to be a secondary manifestation of depression. With increased knowledge, a two-way relationship between sleep disturbances and depression has emerged, namely, sleep issues are predictors of prodromal symptoms rather than an epiphenomenon of sadness [9, 15]. Researchers have not clearly determined whether depression and sleep disturbances have a joint effect on the risk of mortality in cancer survivors.

The purpose of this study was to examine the independent and joint associations between depressive symptoms and sleep disturbances with all-cause, cancer-specific, and noncancer mortality in a nationally representative sample of cancer survivors in the USA. Our findings may encourage cancer survivors, healthcare professionals, and policymakers to increase awareness and promote targeted support to improve patients’ prognoses.

## Methods

### Study population

This prospective cohort study included a nationally representative sample from the National Health and Nutrition Examination Survey (NHANES), which is a biannual and ongoing series of surveys to track the health and nutritional status of the US population, and used a complex, multistage, probability sampling design, with oversampling of various subpopulations to increase estimate accuracy. Participants were selected through a four-stage probability sampling design with primary sampling units selected at the county level, census tract level, and household level in the 50 states and the District of Columbia. Considering the complicated survey design, including oversampling, survey nonresponse, and poststratification, sample weights are provided. All the NHANES protocols used were approved by the Centers for Disease Control and Prevention National Center for Health Statistics Ethics Review Board, and all participants provided written consent after being fully informed. Each participant was invited to participate in a face-to-face interview, a series of physical examinations, and laboratory tests in a mobile examination facility. This study included 2947 cancer survivors aged 20 years or older who participated in the NHANES (2007–2018) survey, and a flowchart of the survivor inclusion process is shown in Fig. S1 (Additional file 1). Informed consent and institutional review board approval were not necessary for the current investigation because we used published data sets from the NHANES that included no personally identifiable information. This prospective cohort study followed the Strengthening in the Reporting of Observational Studies in Epidemiology (STROBE) reporting guidelines.

### Assessment of *cancer*, depressive symptoms, and sleep disturbances

Data on the cancer diagnosis and the number of types, including age at diagnosis, and up to three recorded diagnoses, were gathered from self-reported cancer assessments. Cancer survivors were defined as individuals who provided an affirmative response to the question, “Have you ever been told by a doctor or other health professional that you had cancer or a malignancy of any kind?” and were asked, “What kind of cancer was it?” and “How old were you when cancer was first diagnosed?” The number of years since the first cancer diagnosis was calculated as the difference between the participant’s current age and the age at which they were first diagnosed with cancer.

The Patient Health Questionnaire-9 (PHQ-9) was used to measure the severity of depressive symptoms in cancer survivors during the 2 weeks before the survey, and its validity and performance have been validated in cancer patients [16, 17]. The PHQ-9 consists of nine items on depressive symptoms (lack of interest, depressed mood, trouble sleeping, fatigue, appetite problems, worthlessness, lack of concentration, psychomotor agitation or retardation, and suicidal thoughts). Each item is scored on a scale ranging from 0 (not at all) to 3 (almost daily), adding to a total score ranging from 0 to 27, with higher PHQ-9 scores indicating more severe depressive symptoms. Depressive symptom categories were defined as none (score, 0–4), mild (score, 5–9), or moderate-severe (score, ≥ 10) [18]. Individuals with a total score ≥ 10 points were considered to suffer from major depression; the sensitivity of this threshold was 88% and the specificity was 88% [18].

Survivors’ sleep disturbances in the 2 weeks before the survey were assessed through the self-report question, “How often have you been bothered by trouble falling or staying asleep, or sleeping too much over the last 2 weeks?”, with response options of “not at all,” “several days,” “more than half the days,” and “nearly every day”. Survivors who responded "not at all" were considered to have no sleep disturbances; otherwise, they were considered to have sleep disturbances [19].

### Mortality ascertainment

The primary outcomes included all-cause mortality, cancer-specific mortality, and noncancer mortality. We used the NHANES public-use linked mortality file as of December 31, 2019, which was linked to the National Death Index (NDI) using a probabilistic matching method, to ascertain the mortality status of the follow-up population. The primary cause of death was recorded using the International Statistical Classification of Diseases and Related Health Problems, Tenth Revision (ICD-10). All-cause mortality is defined as death from all causes; cancer-specific mortality is defined as ICD-10 codes C00–C97; deaths from other causes are referred to as noncancer mortality. The number of months from the interview date to the date of death or, for individuals who did not suffer an event, through December 31, 2019, was defined as the follow-up period.

### Covariates

The choice of covariates was made using previous literature and substantive reasoning. Sociodemographic data on age, sex, race and ethnicity, educational level, marital status, the family income to poverty ratio, and work status were gathered using a standardized questionnaire. Participants self-reported their racial and ethnic backgrounds using the National Center for Health Statistics categories of Mexican American, other Hispanic, non-Hispanic White, non-Hispanic Black, and other races or ethnicities. Other races or ethnicities include American Indian/Alaskan Native/Pacific Islander, Asian, and multiracial. The categories for education level were as follows: “less than high school diploma,” “high school diploma or general equivalency diploma,” and “some college or above.” The four categories of married, never married, living with a partner, and other (including widowed, divorced, or separated) were used to classify people’s marital statuses. The family income to poverty ratio was divided into three groups: 1.30 or less, 1.31 to 3.50, and more than 3.50 [20]. The following work statuses were created using the Occupation Questionnaire: nonemployed (including unemployed individuals, retirees, students, and individuals who are not actively looking for work), part-time (working 1–34 h per week), and full-time (working 35 h per week) [21]. Sleep duration was assessed using the self-report question “How much sleep do you get (in hours)?”. Diabetes/hypertension/hypercholesterolemia was self-reported by participants who had been diagnosed by a doctor or other health professional.

The NHANES Prescribing Information Document was consulted for information regarding antidepressant use. During household interviews, data on medication use were gathered. Participants were asked about medication use in the past 30 days. When participants said “yes,” they were requested to present all medication containers or, if none were accessible, to report the name of the medication. The Lexicon Plus database was used to process and classify all prescription medication data. “Psychotherapeutic medications” was the first level category, while “antidepressants” was the second. The use of at least one antidepressant within the previous 30 days was considered antidepressant use in our study.

### Statistical Analysis

A complex sampling design and sampling weights were considered to ensure that the results were nationally representative, and all analyses accounted for the unequal probability of selection, oversampling of specific subpopulations, and nonresponse adjustments by the NHANES analytic guidelines [22]. Data were analyzed using R version 4.3.0. A two-sided *P* < 0.05 was considered to indicate statistical significance.

The baseline characteristics of survivors with varying degrees of depressive symptoms and sleep disturbances are described. Hazard ratios (HRs) and 95% CIs for the correlations of single depressive symptoms or sleep disturbances (adjusted for covariates that did not include each other) with all-cause, cancer-specific, and noncancer mortality were estimated using multivariable Cox proportional hazards regression models. Participants were divided into groups based on depressive symptoms and sleep disturbances to estimate mortality risks and examine joint associations using multivariable Cox proportional hazards regression models adjusted for the same set of covariates. Final-stage multivariable models were adjusted for age, sex, race and ethnicity, educational attainment, marital status, the family income to poverty ratio, work status, diabetes status, hypertension status, hypercholesterolemia status, NHANES cycles, number of cancer types, number of years since the first cancer diagnosis, use of antidepressants, and sleep duration. In addition, subgroup analyses were performed according to age, sex, educational attainment, work status, and use of antidepressants using Cox proportional hazards regression models.

Several sensitivity analyses were conducted to assess the robustness of our findings. First, we performed a sensitivity analysis and excluded participants who died during the initial 2-year follow-up to reduce the likelihood of reverse causation [23]. Second, because of the high cancer mortality rate among Black individuals, we conducted a sensitivity analysis excluding non-Hispanic Black participants [24]. Third, to test the effect of missing variables, multiple interpolation was used to infer all missing independent variables [25].

Mediation studies were performed to determine whether sleep disturbances mediated the association between the exposure variable (depressive symptoms) and the outcome (mortality). In our analysis, thousands of bootstraps were used. The results display p values for mediated effects, the proportion of mediating effects, and indirect pathway effect sizes.

## Results

### Participant characteristics and trends in the prevalence of depressive symptoms and sleep disturbances

**Fig. 1:**
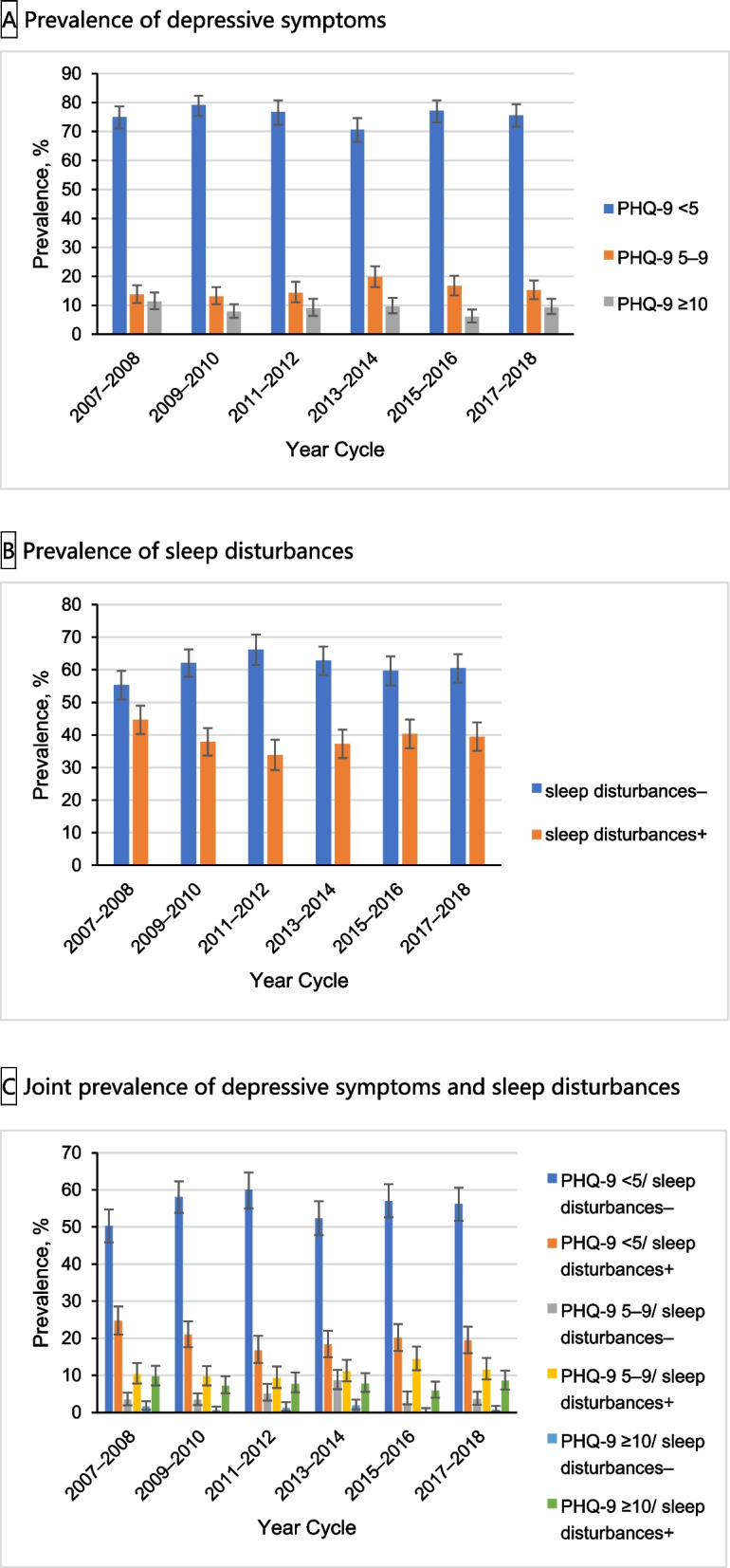
Trends in prevalence of depressive symptoms and sleep disturbances among US cancer survivors aged 20 years or older, National Health and Nutrition Examination Survey 2007–2018. **A** Prevalence of depressive symptoms. **B** Prevalence of sleep disturbances. **C** Joint prevalence of depressive symptoms and sleep disturbances. Abbreviations: PHQ-9, Patient Health Questionnaire-9. Data were weighted to be nationally representative. Error bars indicate 95% CIs

After excluding 421 survivors with missing data for the PHQ-9 and sleep disturbances, a total of 2947 individuals were analyzed. The baseline characteristics of the excluded and included participants are shown in Table S1 (Additional file 2). Of the 2947 cancer survivors (weighted population 21,003,811; weighted mean [SD] age, 62.7 [14.0] years; 1957 non-Hispanic White [85.9%]) in the study cohort, 1550 participants (56.3%) were female. The characteristics of the cancer survivors categorized according to their PHQ-9 scores are presented in Table 1. Cancer survivors with PHQ-9 scores ≥ 5 appeared to be more likely to be female, of a lower socioeconomic status, and to live alone. Cancer survivors with PHQ scores ≥ 5 were more likely to have sleep disturbances. Trends in the prevalence of depressive symptoms and sleep disturbances among cancer survivors aged 20 years or older in the USA are shown in Fig. 1. From 2007–2008 to 2017–2018, the prevalence of PHQ-9 scores (5–9) ranged from 13.1 to 19.8%, the prevalence of PHQ-9 scores (≥ 10) ranged from 6.1 to 11.3%, and the prevalence of sleep disturbances ranged from 33.8 to 44.7%. The joint analysis of the prevalence of depressive symptoms and sleep disturbances showed a low prevalence of participants with PHQ scores (≥ 5) without sleep disturbances, ranging from 2.4 to 8.6% for those with PHQ-9 scores (5–9) without sleep disturbances, and from 0.3 to 1.9% for those with PHQ-9 scores (≥ 10) without sleep disturbances.

**Table 1:** Sample size^a^ and participant characteristics for depressive symptoms and sleep disturbances among US cancer survivors aged 20 years or older, National Health and Nutrition Examination Survey 2007–2018

Characteristics	Participants stratified by PHQ-9 depression score, No. (Weighted %)^b^			
	All	PHQ-9 score 0–4	PHQ-9 score 5–9	PHQ-9 score ≥ 10
Overall	2947 (100)	2154 (75.6)	478 (15.7)	315 (8.7)
Age, years				
20–69	1552 (63.1)	1052 (61.3)	267 (63.0)	233 (78.4)
≥ 70	1395 (36.9)	1102 (38.7)	211 (37.0)	82 (21.6)
Sex				
Male	1397 (43.7)	1123 (47.6)	177 (32.4)	97 (30.6)
Female	1550 (56.3)	1031 (52.4)	301 (67.6)	218 (69.4)
Race and ethnicity				
Mexican American	198 (2.6)	130 (2.4)	29 (2.5)	39 (5.0)
Other Hispanic	194 (2.5)	131 (2.2)	40 (3.7)	23 (3.1)
Non-Hispanic White	1957 (85.9)	1465 (87.2)	313 (84.9)	179 (76.8)
Non-Hispanic Black	435 (5.3)	318 (4.9)	70 (5.8)	47 (7.7)
Other^c^	163 (3.7)	110 (3.4)	26 (3.1)	27 (7.3)
Educational attainment				
Less than high school graduate	590 (11.3)	388 (9.7)	96 (12.5)	106 (23.2)
High school graduate or general equivalency diploma	657 (20.6)	451 (18.6)	133 (28.0)	73 (25.5)
Some college or above	1698 (68.0)	1314 (71.7)	249 (59.5)	135 (51.2)
Marital status				
Married	1663 (62.7)	1304 (66.3)	233 (53.7)	126 (47.1)
Never married	192 (6.1)	118 (5.5)	40 (7.6)	34 (8.3)
Living with partner	92 (3.2)	57 (2.9)	27 (5.2)	8 (2.7)
Other^d^	996 (28.0)	671 (25.2)	178 (33.5)	147 (41.9)
Family poverty income ratio				
≤ 1.3	669 (13.7)	384 (10.1)	134 (18.4)	151 (36.2)
> 1.3 to 3.5	1094 (33.0)	801 (31.1)	195 (40.1)	98 (36.4)
> 3.5	935 (45.6)	785 (50.9)	114 (34.8)	36 (18.3)
Work status				
Nonemployed	2100 (61.3)	1479 (58.6)	369 (68.8)	252 (71.8)
Part time (1–34 h/wk)	295 (11.4)	230 (11.8)	46 (12.2)	19 (6.9)
Full time (≥ 35 h/wk)	543 (26.9)	438 (29.3)	62 (18.6)	43 (20.8)
Diabetes				
No	2325 (83.2)	1725 (84.4)	365 (78.4)	235 (80.6)
Yes	621 (16.8)	429 (15.6)	113 (21.6)	79 (19.2)
Hypertension				
No	1229 (47.9)	933 (50.2)	181 (40.1)	115 (41.9)
Yes	1713 (52.0)	1217 (49.7)	296 (59.6)	200 (58.1)
Hypercholesterolemia				
No	1277 (44.2)	951 (45.2)	203 (41.6)	123 (40.1)
Yes	1531 (52.4)	1122 (52.0)	247 (55.1)	162 (51.3)
Number of cancer types				
1	2645 (89.8)	1947 (90.5)	425 (88.3)	273 (86.1)
2	267 (9.0)	185 (8.5)	47 (10.1)	35 (11.7)
≥ 3	35 (1.2)	22 (1.0)	6 (1.6)	7 (2.2)
Age at cancer first diagnosed, years				
< 40	562 (23.3)	351 (20.8)	110 (27.4)	101 (37.0)
40–60	1181 (44.7)	847 (45.0)	193 (43.1)	141 (45.0)
> 60	1183 (31.6)	944 (33.8)	174 (29.4)	65 (16.4)
Use of antidepressants				
No	2270 (74.6)	1802 (81.1)	313 (60.0)	155 (44.6)
Yes	645 (24.6)	332 (18.3)	157 (39.2)	156 (52.7)
Daily sleep duration, h				
< 7	917 (27.5)	573 (22.8)	196 (38.9)	148 (47.2)
7–9	1546 (57.2)	1239 (62.6)	200 (45.0)	107 (32.9)
≥ 9	470 (14.6)	336 (14.0)	79 (15.8)	55 (18.1)
Sleep disturbances				
No	1784 (61.2)	1623 (73.7)	127 (28.4)	34 (11.7)
Yes	1163 (38.8)	531 (26.3)	351 (71.6)	281 (88.3)

*Abbreviations*: *PHQ-9* Patient Health Questionnaire-9, *h/wk* hours per week

^a^Weighted to be nationally representative. The sum of weighted percentages may not equal 100% due to missing data

^b^The number of participants is unweighted. All percentage estimates are weighted

^c^Other includes any other race or ethnicity other than Mexican American, other Hispanic, non-Hispanic White, or non-Hispanic Black

^d^Including widowed, divorced, or separated individuals

### Individual associations between PHQ-9 scores or sleep disturbances and mortality

During the median follow-up of 69 months (interquartile range, 37–109 months), only one participant failed to complete follow-up, and a total of 686 deaths occurred: 240 participants died of cancer, 146 died of heart disease, and 300 died of other causes. Separate analyses that included only PHQ-9 scores or sleep disturbances showed that cancer survivors with higher PHQ-9 scores had greater risks of all-cause and noncancer mortality (Table 2), but sleep disturbances alone were not associated with an increased risk of mortality. After adjusting for covariates, compared with individuals with PHQ-9 scores (0–4), the HRs for all-cause mortality for individuals with PHQ-9 scores (5–9) and PHQ-9 scores (≥ 10) were 1.28 (1.03–1.59) and 1.37 (1.04–1.80), respectively; the HRs for cancer-specific mortality were 1.42 (0.99–2.04) and 1.17 (0.71–1.94), respectively; and the HRs for noncancer mortality were 1.21 (0.91–1.59) and 1.45 (1.01–2.10), respectively. In addition, the risks of all-cause and noncancer mortality increased by 3% and 4%, respectively, for each 1-point increase in the PHQ-9 score.

**Table 2:** Association of PHQ-9 score and sleep disturbances with all-cause, cancer, and noncancer mortality among US cancer survivors aged 20 years or older, National Health and Nutrition Examination Survey 2007–2018

				**Hazard ratio (95% CI)**	
**Mortality outcome**	**Death/No**	**Weighted death (%)**	**Age adjusted**^**a**^	**MV model 1**^**b**^	**MV model 2**^**b, c**^
**All causes**					
PHQ-9 depression score					
0–4	491/2154	2,454,086 (15.5)	1 [Reference]	1 [Reference]	1 [Reference]
5–9	126/478	647,945 (19.7)	1.42 (1.16–1.72)	1.32 (1.07–1.62)	1.28 (1.03–1.59)
≥ 10	69/315	304,281 (16.6)	1.84 (1.42–2.37)	1.44 (1.09–1.90)	1.37 (1.04–1.80)
Per 1 point increase in PHQ-9	NA	NA	1.05 (1.04–1.06)	1.04 (1.02–1.05)	1.03 (1.01–1.05)
Sleep disturbances					
No	436/1784	2,191,440 (17.1)	1 [Reference]	1 [Reference]	1 [Reference]
Yes	250/1163	1,214,871 (14.9)	1.06 (0.90–1.24)	0.99 (0.84–1.17)	1.02 (0.86–1.22)
**Cancer**					
PHQ-9 depression score					
0–4	168/2154	844,742 (5.3)	1 [Reference]	1 [Reference]	1 [Reference]
5–9	47/478	246,781 (7.5)	1.47 (1.07–2.04)	1.38 (0.98–1.93)	1.42 (0.99–2.04)
≥ 10	25/315	90,294 (4.9)	1.54 (1.00–2.36)	1.15 (0.73–1.83)	1.17 (0.71–1.94)
Per 1 point increase in PHQ-9	NA	NA	1.04 (1.01–1.06)	1.02 (0.99–1.05)	1.02 (0.99–1.05)
Sleep disturbances					
No	152/1784	778,817 (6.1)	1 [Reference]	1 [Reference]	1 [Reference]
Yes	88/1163	403,001 (4.9)	0.99 (0.76–1.30)	0.90 (0.68–1.19)	0.94 (0.70–1.27)
**Noncancer**					
PHQ-9 depression score					
0–4	323/2154	1,609,343 (10.1)	1 [Reference]	1 [Reference]	1 [Reference]
5–9	79/478	401,164 (12.2)	1.37 (1.07–1.75)	1.28 (0.98–1.67)	1.21 (0.91–1.59)
≥ 10	44/315	213,987 (11.7)	2.05 (1.49–2.82)	1.69 (1.20–2.38)	1.45 (1.01–2.10)
Per 1 point increase in PHQ-9	NA	NA	1.06 (1.04–1.09)	1.05 (1.03–1.07)	1.04 (1.01–1.06)
Sleep disturbances					
No	284/1784	1,412,623 (11.0)	1 [Reference]	1 [Reference]	1 [Reference]
Yes	162/1163	811,871 (10.0)	1.10 (0.90–1.33)	1.06 (0.86–1.30)	1.08 (0.87–1.34)

*Abbreviations*: *PHQ-9* Patient Health Questionnaire-9, *MV* multivariable

^a^Adjusted for age

^b^Multivariable model adjusted for age, sex (male/female), race and ethnicity (Mexican American, other Hispanic, non-Hispanic White, non-Hispanic Black, other race or ethnicity [including American Indian/Alaska Native/Pacific Islander, Asian, multiracial]), educational attainment (< high school graduate, high school graduate or general equivalency diploma, ≥ Some college), marital status (married, never married, living with partner, other [including widowed, divorced, separated individuals]), family poverty income ratio (≤ 1.3, 1.3–3.5, > 3.5), work status (nonemployed, part time [1–34 h/wk], full time [≥ 35 h/wk]), and National Health and Nutrition Examination Survey cycles (2007–2008, 2009–2010, 2011–2012, 2013–2014, 2015–2016, 2017–2018)

^c^Additionally adjusted for diabetes (yes/no), hypertension (yes/no), hypercholesterolemia (yes/no), the number of cancer types (1, 2, ≥ 3), the number of years since the first cancer diagnosis, use of antidepressants (yes/no), and sleep duration

### Joint association of PHQ-9 scores and sleep disturbances with mortality

**Fig. 2:**
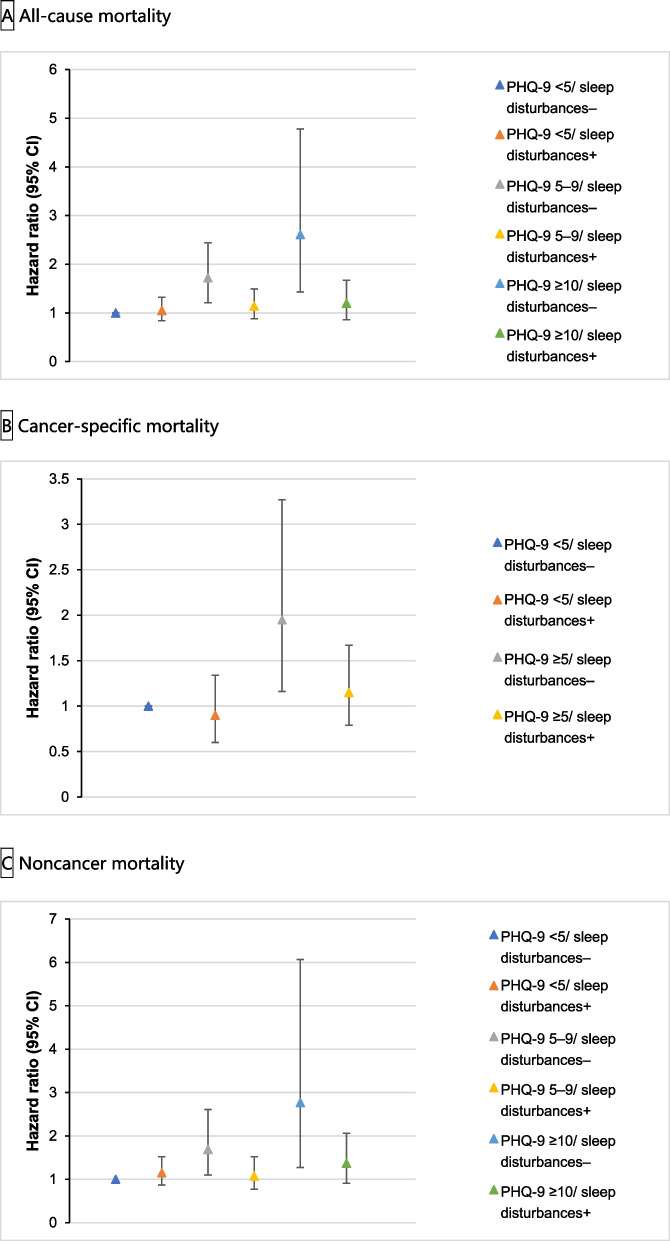
Joint association of PHQ-9 score and sleep disturbances with all-cause, cancer, and noncancer mortality among US cancer survivors aged 20 years or older, National Health and Nutrition Examination Survey 2007–2018. **A** All-cause mortality. **B** Cancer-specific mortality. **C** Noncancer mortality. Abbreviations: PHQ-9, Patient Health Questionnaire-9. Hazard ratios (solid symbols) with 95% CIs (error bars) of joint categories of depression and sleep disturbances for **A** all-cause, **B** cancer-specific, and **C** noncancer mortality were estimated using multivariable Cox regression models adjusted for age, sex, race and ethnicity, educational attainment, marital status, family poverty income ratio, work status, National Health and Nutrition Examination Survey cycles, diabetes, hypertension, hypercholesterolemia, the number of cancer types, the number of years since the first cancer diagnosis, use of antidepressants, and sleep duration

We attempted to perform a joint analysis of depression, sleep disturbances, and mortality in cancer survivors. Surprisingly, in the joint analysis, the combination of a PHQ-9 score (≥ 5) and no sleep disturbances was associated with the risks of all-cause mortality, cancer-specific mortality, and noncancer mortality (Fig. 2 and Table 3). Specifically, compared with individuals with a PHQ-9 score of 0–4 and no sleep disturbances, individuals with a PHQ-9 score of 5–9 and no sleep disturbances had HRs of 1.72 (1.21–2.44) and 1.69 (1.10–2.61) for all-cause mortality and noncancer mortality, respectively; individuals with a PHQ-9 score ≥ 10 and no sleep disturbances had HRs of 2.61 (1.43–4.78) and 2.77 (1.27–6.07) for all-cause and noncancer mortality, respectively; and HRs for cancer-specific mortality in individuals with a PHQ-9 score ≥ 5 and no sleep disturbances were 1.95 (1.16–3.27).

**Table 3:** Joint association of PHQ-9 score and sleep disturbances with all-cause, cancer, and noncancer mortality among US cancer survivors aged 20 years or older, National Health and Nutrition Examination Survey 2007–2018

				**Hazard ratio (95% CI)**	
**Mortality outcome**	**Sleep disturbances**	**Death/No**	**Weighted death (%)**	**MV model 1**^**a**^	**MV model 2**^**a, b**^
**All causes**					
PHQ-9 score 0–4	No	379/1623	1,872,111 (16.0)	1 [Reference]	1 [Reference]
	Yes	112/531	581,975 (13.9)	0.97 (0.78–1.21)	1.05 (0.84–1.32)
PHQ-9 score 5–9	No	44/127	256,511 (27.4)	1.80 (1.30–2.50)	1.72 (1.21–2.44)
	Yes	82/351	391,433 (16.6)	1.14 (0.88–1.46)	1.14 (0.88–1.49)
PHQ-9 score ≥ 10	No	13/34	62,818 (29.3)	2.70 (1.52–4.79)	2.61 (1.43–4.78)
	Yes	56/281	241,463 (14.9)	1.28 (0.94–1.74)	1.20 (0.86–1.67)
**Cancer**					
PHQ-9 score 0–4	No	132/1623	681,292 (5.8)	1 [Reference]	1 [Reference]
	Yes	36/531	163,451 (3.9)	0.83 (0.56–1.22)	0.90 (0.60–1.34)
PHQ-9 score ≥ 5	No	20/161	97,525 (8.5)	1.84 (1.12–3.02)	1.95 (1.16–3.27)
	Yes	52/632	239,550 (6.0)	1.10 (0.77–1.55)	1.15 (0.79–1.67)
**Noncancer**					
PHQ-9 score 0–4	No	247/1623	1,190,819 (10.2)	1 [Reference]	1 [Reference]
	Yes	76/531	418,525 (10.0)	1.06 (0.81–1.39)	1.15 (0.87–1.52)
PHQ-9 score 5–9	No	29/127	172,879 (18.5)	1.84 (1.22–2.76)	1.69 (1.10–2.61)
	Yes	50/351	228,285 (9.7)	1.11 (0.80–1.53)	1.08 (0.77–1.52)
PHQ-9 score ≥ 10	No	8/34	48,926 (22.8)	3.27 (1.58–6.80)	2.77 (1.27–6.07)
	Yes	36/281	165,061 (10.2)	1.54 (1.06–2.25)	1.37 (0.91–2.06)

*Abbreviations*: *PHQ-9* Patient Health Questionnaire-9, *MV* multivariable

^a^Multivariable model adjusted for age, sex (male/female), race and ethnicity (Mexican American, other Hispanic, non-Hispanic White, non-Hispanic Black, other race or ethnicity [including American Indian/Alaska Native/Pacific Islander, Asian, multiracial]), educational attainment (< high school graduate, high school graduate or general equivalency diploma, ≥ Some college), marital status (married, never married, living with partner, other [including widowed, divorced, separated individuals]), family poverty income ratio (≤ 1.3, 1.3–3.5, > 3.5), work status (nonemployed, part time [1–34 h/wk], full time [≥ 35 h/wk]), and National Health and Nutrition Examination Survey cycles (2007–2008, 2009–2010, 2011–2012, 2013–2014, 2015–2016, 2017–2018)

^b^Additionally adjusted for diabetes (yes/no), hypertension (yes/no), hypercholesterolemia (yes/no), the number of cancer types (1, 2, ≥ 3), the number of years since the first cancer diagnosis, use of antidepressants (yes/no), and sleep duration

### Mediation analysis of the effects of sleep disturbances on associations of the PHQ-9 score with mortality

A mediation analysis of the effects of sleep disturbances on the relationship between PHQ-9 scores and mortality was performed to clarify whether sleep disturbances mediate the relationship between depressive symptoms and mortality, and the results showed that sleep disturbances were not a mediator between the relationship between PHQ-9 scores and mortality (Additional file 3: Table S2).

### Subgroup analyses

The results of the subgroup analyses are displayed in Fig. S2 (Additional file 4). Among participants with a PHQ-9 score ≥ 5 and no sleep disturbances, participants aged 70 years or older (HR, 2.36; 95% CI, 1.64–3.37), both male (HR, 2.00; 95% CI, 1.28–3.13) and female (HR, 1.72; 1.10–2.70) participants, participants with less than some college education (HR, 1.92; 95% CI, 1.25–2.94), participants with some college education or above (HR, 1.98; 95% CI, 1.23–3.17), nonemployed participants (HR, 1.98; 95% CI, 1.44–2.73), and participants who did not use antidepressants (HR, 2.37; 95% CI, 1.59–3.53) had increased all-cause mortality risks. Participants aged 70 years or older (HR, 3.60; 95% CI, 1.94–6.68), participants with some college education or above (HR, 3.22; 95% CI, 1.56–6.67), and nonemployed participants (HR, 2.25; 95% CI, 1.33–3.83) had increased cancer-specific mortality risks. Participants aged 70 years or older (HR, 1.98; 95% CI, 1.27–3.10), male participants (HR, 2.19; 95% CI, 1.27–3.78), participants with less than some college education (HR, 2.41; 95% CI, 1.44–4.04), nonemployed participants (HR, 1.87; 95% CI, 1.24–2.80), and participants who did not use antidepressants (HR, 2.72; 95% CI, 1.66–4.44) had increased noncancer mortality risks.

### Sensitivity analyses

Tables S3–5 (Additional file 5–7) summarize the results of the sensitivity analyses. After excluding participants who died during the first 2 years of follow-up (unweighted *n* = 2790), cancer survivors with a PHQ-9 score ≥ 10 and no sleep disturbances still had increased all-cause (HR, 2.89; 95% CI, 1.43–5.85) and noncancer mortality risks (HR, 3.49; 95% CI, 1.49–8.22). Second, after excluding non-Hispanic Black participants (unweighted *n* = 2512), cancer survivors with a PHQ-9 score of 5–9 and no sleep disturbances and cancer survivors with a PHQ-9 score ≥ 10 and no sleep disturbances still had increased all-cause and noncancer mortality risks. Third, the results remain similar after multiple interpolations for all missing independent variables (Additional file 8: Table S6).

## Discussion

In this nationally representative cohort of cancer survivors in the USA, we first examined the prevalence of depressive symptoms and sleep disturbances among cancer survivors. Our findings indicated that more than one-third of participants reported having sleep disturbances, and more than 20% reported having at least mild depressive symptoms, with close to 10% having moderate to severe depressive symptoms. Our findings revealed an independent effect of depressive symptoms on mortality, whereas sleep disturbances alone did not exhibit this effect. We attempted to perform a joint analysis of depression, sleep disturbances, and mortality in cancer survivors. Interestingly, in the joint analysis, an association between depressive symptoms and an increased risk of mortality was observed only in cancer survivors without sleep disturbances, whereas no such association was observed in cancer survivors with sleep disturbances. Notably, among cancer survivors without sleep disturbances, individuals with a PHQ-9 score ≥ 5 had increased risks of all-cause mortality, cancer-specific mortality, and noncancer mortality, particularly individuals with a PHQ-9 score ≥ 10.

To our knowledge, this study is the first to examine the separate and combined relationships of depressive symptoms and sleep disturbances with mortality outcomes in a sample of cancer survivors from throughout the USA. Depression and sleep disturbances are important research targets for cancer survivors given their high prevalence rates and possible impact on prognosis. Much of the previous research has focused exclusively on depression, with little consideration of its joint effect with sleep disturbances. Consistent with previous studies [26, 27], our study showed that depressive symptoms in cancer survivors are associated with a higher mortality rate and a generally worse prognosis, suggesting that depression may influence disease progression. In addition, because of the high prevalence of sleep disturbances among cancer patients [3] and the possible role of sleep disturbances in tumor progression [12], we were also interested in the association between sleep disturbances and the mortality risk and whether the extent of the association between depressive symptoms and mortality risk varied by sleep disturbances. Our study showed that although single sleep disturbances were not associated with the mortality risk, surprisingly, PHQ-9 scores (≥ 5) without sleep disturbances were associated with increased all-cause, cancer-specific, and noncancer mortality risks, whereas PHQ-9 scores (≥ 5) with sleep disturbances were not. We also performed mediation analyses, and the results showed that sleep disturbances were not a mediator of the relationship between PHQ-9 scores and mortality. Therefore, we attribute the findings of this study to the heterogeneity of depression [28]. Specifically, depression without sleep disturbances may be a subtype of depression associated with a poorer prognosis among cancer survivors. This result suggests that the absence of sleep disturbances, although low in prevalence among cancer survivors with depressive symptoms, is associated with a high risk of mortality in this group. Among cancer survivors, depression screening is needed via a simple PHQ-9 questionnaire, and among individuals with a self-reported absence of sleep disturbances, individuals with a PHQ-9 score ≥ 5 should receive more attention, especially those with a PHQ-9 score ≥ 10.

The relationship between sleep and the mortality risk is debatable and appears to differ between groups with and without tumors. In the total population, sleep disturbance and insufficient or excessive sleep are associated with an increased risk of death [29, 30]. Previous meta-analyses and systematic evaluations have provided mixed results regarding poor sleep and the all-cause mortality risk [31, 32]. Yin and colleagues concluded that both short and long sleep durations (less or more than 7 h/day) were associated with the all-cause mortality risk, with a pooled relative risk (RR) of 1.06 (95% CI, 1.04–1.07) per 1-h decrease in the population with a short sleep duration and 1.13 (95% CI, 1.11–1.15) per 1-h increase in the population with a long sleep duration [31]. In contrast, Kwok et al. concluded that long sleepers (> 8 h/day), but not short sleepers (< 7 h/day), had a greater risk of all-cause mortality, with an 11-h RR of 1.47 and a 95% CI of 1.33–1.64; sleep disturbance was not associated with increased mortality, with an RR of 1.03 and 95% CI of 0.93–1.14 [32]. Regarding the community of people who had survived tumors, Sun and colleagues found that getting sufficient sleep (6 h or more per day) was strongly connected with a lower chance of death in cancer patients [33]. Since these previous studies seemed to show a relationship between the sleep duration and mortality, our study adjusted for sleep duration, and the results still showed a high risk of mortality in patients with depressive symptoms and no self-perceived sleep disturbances. Additionally, evidence has shown that circadian rhythms play a role in carcinogenesis [12, 34–37], but the complex links involved are controversial, and more research is needed. Recent preclinical studies have shown that cancer cells are affected by melatonin during sleep and may “wake up” and become even more active than during the day [12, 13]. Interestingly, our study revealed that cancer survivors with no self-perceived sleep disturbances and a PHQ-9 score ≥ 5 had increased risks of both cancer-specific and all-cause mortality, whereas those with self-perceived sleep disturbances did not have an increased risk of mortality. This finding seems to indirectly indicate the cancer-promoting effects of sleep among cancer survivors in the context of depression, a highly inflammatory condition [38]. However, the reasons for this finding remain to be explored in greater depth to determine the complex links between depression, sleep, and cancer.

The joint effects of depression and sleep issues on the mortality risk in the general population have also rarely been studied. A prospective cohort study analyzing the data of 25,978 adults from the NHANES 2005–2014 cycle [39] revealed no joint effects of depression and sleep disturbances on total mortality and showed that sleep disturbances were significantly associated with total mortality, with an HR of 1.49 and a 95% CI of 1.28–1.72. This result contradicts our findings and points to the uniqueness of depression and sleep in cancer patients. As a result, our findings may therefore encourage cancer survivors, medical professionals, and decision-makers to increase awareness to promote tailored support, which has the potential to improve cancer survivors’ prognoses.

Previous studies have reported that excessive sleep increases the risk of total mortality in older adults [39, 40]. According to our subgroup analyses, we found that older survivors with a PHQ-9 score ≥ 5 and no self-perceived sleep disturbances had increased risks of all-cause, cancer-specific, and noncancer mortality after adjusting for the sleep duration, suggesting that healthcare professionals may need to focus on this group. In addition, we found that a PHQ-9 score ≥ 5 and the absence of sleep disturbances were more strongly associated with noncancer mortality in male survivors than in female survivors. This finding is consistent with previously reported findings on depressive symptoms and mortality, which suggested a stronger association between depressive symptoms and mortality in men than in women [41, 42]. These findings suggest that sex may be an influential factor, emphasizing the value of considering sex differences in future studies. Interestingly, among survivors with a PHQ-9 score ≥ 5 and no sleep disturbances, no use of antidepressant medications was more strongly associated with mortality than antidepressant medication use. The use of antidepressants can affect sleep, which varies depending on the type of antidepressant (e.g., some medications suppress rapid eye movement sleep, thereby affecting sleep structure) [43]. This result may suggest that the effect of sleep on mortality in depressed cancer survivors may be related to sleep quality/sleep architecture and not just sleep duration/ “disturbance.” In addition, sleep quality/sleep architecture may be associated with pro-tumor in the context of depression, which is a highly inflammatory condition. However, due to the lack of standardized assessment tools capable of capturing the multidimensional properties of sleep (e.g., sleep questionnaires with established psychometric properties or polysomnographic monitoring), the specific sleep multidimensional components controlling tumor progression remain unclear. Therefore, further studies are necessary to thoroughly clarify the association between sleep and tumor growth, thus making it possible to adopt non-medical sleep interventions to improve survival outcomes in tumor patients.

### Strengths and limitations

The clear strengths of this study include the use of a well-established nationally representative sample of cancer survivors from the USA, the population-based design, and adjustment for a range of potential confounders, as well as the performance of stratified analyses with sufficient statistical efficacy, which allows the generalization of the findings at the population level.

Some limitations in the current study should also be noted. First, due to data limitations, the multidimensional nature of sleep disturbances was not investigated using a standardized assessment tool capable of capturing this, and despite adjustments for self-reported sleep duration, it may be subject to bias. Second, depressive symptoms were assessed using the PHQ-9, which may not be a perfect indicator of depressive status. Third, this study assessed depressive symptoms and sleep disturbance status in the 2 weeks before the survey and did not reflect historical diagnosis. However, they were measured during the same period, ensuring that the two factors were consistent at the point in time. Fourth, long-term use of antidepressants is a significant potential confounder of mortality, but we did not have access to participants’ long-term use of antidepressants. In addition, following the baseline data collection, we could not obtain information regarding modifications to the participant’s physical condition. Finally, because the NHANES is not a dedicated database for cancer patients, data on cancer stages and treatment were not collected.

## Conclusions

In this prospective cohort study including a nationally representative sample of cancer survivors in the USA, the combination of the PHQ-9 score (≥ 5) and the absence of self-perceived sleep disturbances was associated with greater risks of all-cause mortality, cancer-specific mortality, and noncancer mortality, particularly in individuals with a PHQ-9 score ≥ 10. These findings have important public health implications and suggest the need for depression screening, sleep investigation, and targeted treatment for cancer survivors.

### Supplementary Information

Additional file 1: Figure S1. Flowchart for Screening and Enrollment of Study Participants.

Additional file 2: Table S1. Characteristics of Excluded and Included Participants.

Additional file 3: Table S2. Mediation Analysis of Sleep Disturbances on Associations of PHQ-9 Score with Mortality.

Additional file 4: Figure S2. Adjusted Hazard Ratios for All-cause Mortality, Cancer Mortality, and Noncancer Mortality in PHQ-9 ≥ 5/ Sleep disturbances– Participants Compared with the PHQ-9 < 5/ sleep disturbances–, Stratified by Age, Sex, Educational Attainment, Work Status, and Use of Antidepressants.

Additional file 5: Table S3. Sensitivity Analyses for All-cause Mortality According to PHQ-9 Score and Sleep Disturbances.

Additional file 6: Table S4. Sensitivity Analyses for Cancer-specific Mortality According to PHQ-9 Score and Sleep Disturbances.

Additional file 7: Table S5. Sensitivity Analyses for Noncancer Mortality According to PHQ-9 Score and Sleep Disturbances.

Additional file 8: Table S6. Joint Association of PHQ-9 Score and Sleep Disturbances with All-Cause, Cancer, and Noncancer Mortality Among US Cancer Survivors Aged 20 Years or Older, National Health and Nutrition Examination Survey 2007–2018: Multiple Imputation for Sensitivity Analysis.

## Data Availability

The publicly available datasets used in this study can be accessed at https://www.cdc.gov/nchs/nhanes/index.htm.

## Abbreviations

ASCO: American Society of Clinical Oncology
HR: Hazard ratio
ICD-10: International Statistical Classification of Diseases and Related Health Problems, Tenth Revision
NCCN: National Comprehensive Cancer Network
NDI: National Death Index
NHANES: National Health and Nutrition Examination Survey
PHQ-9: Patient Health Questionnaire 9
RR: Relative risk
STROBE: Strengthening in the Reporting of Observational Studies in Epidemiology
