# Supplementary material for: Association of depressive symptoms and sleep disturbances with survival among US adult cancer survivors
Source: BMC Med. 2024 Jun 5;22:225. doi: 10.1186/s12916-024-03451-7 (PMC11151538; doi:10.1186/s12916-024-03451-7)
Supplement: Supplementary file 1 — Additional file 1: Figure S1. Flowchart for Screening and Enrollment of Study Participants. [file 12916_2024_3451_MOESM1_ESM.docx]

**Fig. S1.** Flowchart for Screening and Enrollment of Study Participants


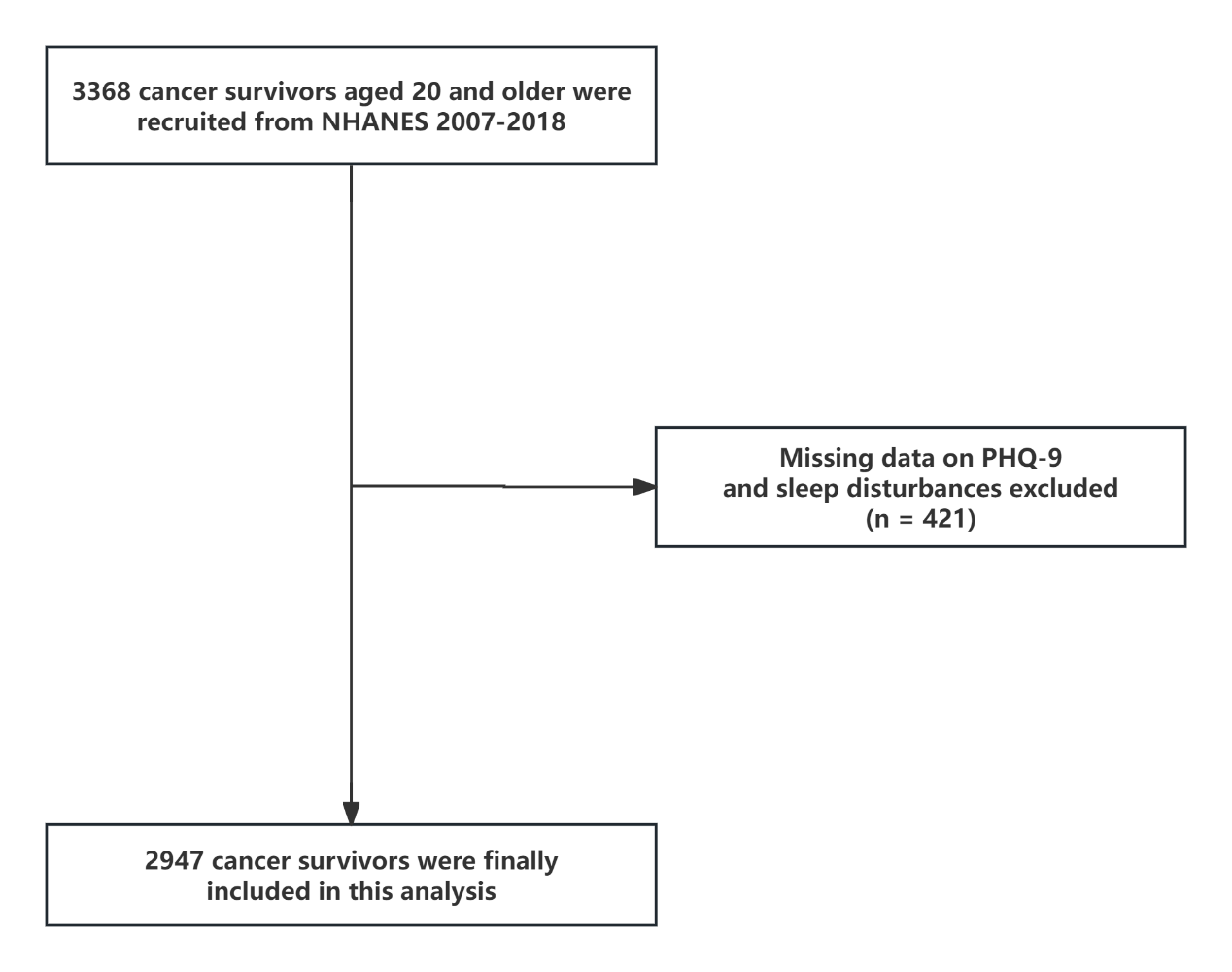


Abbreviations: NHANES, National Health and Nutrition Examination Survey; PHQ-9, Patient Health Questionnaire-9.
