# Supplementary material for: Association of depressive symptoms and sleep disturbances with survival among US adult cancer survivors
Source: BMC Med. 2024 Jun 5;22:225. doi: 10.1186/s12916-024-03451-7 (PMC11151538; doi:10.1186/s12916-024-03451-7)
Supplement: Supplementary file 2 — Additional file 2: Table S1. Characteristics of Excluded and Included Participants. [file 12916_2024_3451_MOESM2_ESM.docx]

**Table S1.** Characteristics of Excluded and Included Participants

| Characteristics | No. (Weighted %)^a^ | |  |
| --- | --- | --- | --- |
|  | Participants excluded (N = 421) | Participants included (N = 2947) | *p* Value |
| Age, y |  |  | <0.001 |
| 20-69 | 170 (49.8) | 1552 (63.1) |  |
| ≥70 | 251 (50.2) | 1395 (36.9) |  |
| Sex |  |  | 0.636 |
| Male | 198 (42.1) | 1397 (43.7) |  |
| Female | 223 (57.9) | 1550 (56.3) |  |
| Race and ethnicity |  |  | 0.005 |
| Mexican American | 37 (3.4) | 198 (2.6) |  |
| Other Hispanic | 32 (3.5) | 194 (2.5) |  |
| Non-Hispanic White | 252 (79.7) | 1957 (85.9) |  |
| Non-Hispanic Black | 62 (6.7) | 435 (5.3) |  |
| Other^b^ | 38 (6.6) | 163 (3.7) |  |
| Educational attainment |  |  | <0.001 |
| Less than high school graduate | 143 (22.8) | 590 (11.3) |  |
| High school graduate or general equivalency diploma | 80 (19.8) | 657 (20.6) |  |
| Some college or above | 196 (57.0) | 1698 (68.0) |  |
| Missing | 2 (0.4) | 2 (0.0) |  |
| Marital status |  |  | 0.001 |
| Married | 198 (50.7) | 1663 (62.7) |  |
| Never married | 33 (7.5) | 192 (6.1) |  |
| Living with partner | 19 (3.8) | 92 (3.2) |  |
| Other^c^ | 169 (37.8) | 996 (28.0) |  |
| Missing | 2 (0.3) | 4 (0.0) |  |
| Family poverty income ratio |  |  | <0.001 |
| ≤1.3 | 124 (18.4) | 669 (13.7) |  |
| ＞1.3 to 3.5 | 119 (24.8) | 1094 (33.0) |  |
| ＞3.5 | 102 (36.3) | 935 (45.6) |  |
| Missing | 76 (20.4) | 249 (7.8) |  |
| Work status |  |  | 0.005 |
| Nonemployed | 339 (72.8) | 2100 (61.3) |  |
| Part time (1-34 h/wk) | 20 (5.6) | 295 (11.4) |  |
| Full time (≥35 h/wk) | 60 (20.8) | 543 (26.9) |  |
| Missing | 2 (0.8) | 9 (0.3) |  |
| Diabetes |  |  | 0.007 |
| No | 333 (85.3) | 2325 (83.2) |  |
| Yes | 87 (14.3) | 621 (16.8) |  |
| Missing | 1 (0.4) | 1 (0.0) |  |
| Hypertension |  |  | 0.911 |
| No | 183 (47.1) | 1229 (47.9) |  |
| Yes | 237 (52.7) | 1713 (52.0) |  |
| Missing | 1 (0.2) | 5 (0.1) |  |
| Hypercholesterolemia |  |  | <0.001 |
| No | 216 (55.7) | 1277 (44.2) |  |
| Yes | 177 (39.0) | 1531 (52.4) |  |
| Missing | 28 (5.3) | 139 (3.4) |  |
| Number of cancer types |  |  | 0.504 |
| 1 | 366 (87.3) | 2645 (89.8) |  |
| 2 | 48 (11.3) | 267 (9.0) |  |
| ≥3 | 7 (1.4) | 35 (1.2) |  |
| Age at cancer first diagnosed, y |  |  | 0.002 |
| <40 | 72 (22.3) | 562 (23.3) |  |
| 40–60 | 126 (35.5) | 1181 (44.7) |  |
| >60 | 216 (40.3) | 1183 (31.6) |  |
| Missing | 7 (1.9) | 21 (0.5) |  |
| Use of antidepressants |  |  | <0.001 |
| No | 291 (72.0) | 2270 (74.6) |  |
| Yes | 112 (23.9) | 645 (24.6) |  |
| Missing | 18 (4.1) | 32 (0.8) |  |
| Daily sleep duration, h |  |  | 0.049 |
| <7 | 117 (21.1) | 917 (27.5) |  |
| 7–9 | 210 (58.2) | 1546 (57.2) |  |
| ≥9 | 90 (19.7) | 470 (14.6) |  |
| Missing | 4 (0.9) | 14 (0.6) |  |

Abbreviations: h/wk, hours per week.

^a^ The number of participants is unweighted. All percentage estimates are weighted to be nationally representative.

^b^ Other includes any other race or ethnicity other than Mexican American, other Hispanic, non-Hispanic White, or non-Hispanic Black.

^c^ Including widowed, divorced, or separated individuals.
