# Supplementary material for: Association of depressive symptoms and sleep disturbances with survival among US adult cancer survivors
Source: BMC Med. 2024 Jun 5;22:225. doi: 10.1186/s12916-024-03451-7 (PMC11151538; doi:10.1186/s12916-024-03451-7)
Supplement: Supplementary file 3 — Additional file 3: Table S2. Mediation Analysis of Sleep Disturbances on Associations of PHQ-9 Score with Mortality. [file 12916_2024_3451_MOESM3_ESM.docx]

**Table S2.** Mediation Analysis of Sleep Disturbances on Associations of PHQ-9 Score with Mortality

|  | Sleep disturbances |  |
| --- | --- | --- |
| PHQ-9 depression score | Estimation% (95% CI) | *p*-value |
| **All causes** |  |  |
| Total effect | -9.28 (-17.40, -2.02) | 0.012 |
| Indirect effect | 3.41 (-1.29, 10.60) | 0.188 |
| Direct effect | -12.69 (-23.01, -4.43) | 0.004 |
| Proportion mediated | -0.31 (-2.19, 0.18) | 0.200 |
| **Cancer** |  |  |
| Total effect | -21.96 (-71.99, 13.13) | 0.30 |
| Indirect effect | 17.58 (-7.53, 57.69) | 0.23 |
| Direct effect | -39.54 (-107.52, 5.57) | 0.10 |
| Proportion mediated | -0.44 (-11.61, 11.86) | 0.47 |
| **Noncancer** |  |  |
| Total effect | -16.63 (-32.55, -3.55) | 0.008 |
| Indirect effect | 4.02 (-4.71, 15.14) | 0.384 |
| Direct effect | -20.65 (-40.55, -6.45) | <0.001 |
| Proportion mediated | -0.21 (-1.74, 0.30) | 0.392 |

Models were adjusted for age, sex (male/female), race and ethnicity (Mexican American, other Hispanic, non-Hispanic White, non-Hispanic Black, other race or ethnicity [including American Indian/Alaska Native/Pacific Islander, Asian, multiracial]), educational attainment (<high school graduate, high school graduate or general equivalency diploma, ≥Some college), marital status (married, never married, living with partner, other [including widowed, divorced, separated individuals]), family poverty income ratio (≤1.3, 1.3–3.5, ＞3.5), work status (nonemployed, part time [1–34 h/wk], full time [≥35 h/wk]), National Health and Nutrition Examination Survey cycles (2007–2008, 2009–2010, 2011–2012, 2013–2014, 2015–2016, 2017–2018), diabetes (yes/no), hypertension (yes/no), hypercholesterolemia (yes/no), the number of cancer types (1, 2, ≥3), the number of years since the first cancer diagnosis, use of antidepressants (yes/no), and sleep duration.
