# Supplementary material for: Association of depressive symptoms and sleep disturbances with survival among US adult cancer survivors
Source: BMC Med. 2024 Jun 5;22:225. doi: 10.1186/s12916-024-03451-7 (PMC11151538; doi:10.1186/s12916-024-03451-7)
Supplement: Supplementary file 4 — Additional file 4: Figure S2. Adjusted Hazard Ratios for All-cause Mortality, Cancer Mortality, and Noncancer Mortality in PHQ-9 ≥ 5/ Sleep disturbances– Participants Compared with the PHQ-9 < 5/ sleep disturbances–, Stratified by Age, Sex, Educational Attainment, Work Status, and Use of Antidepressants. [file 12916_2024_3451_MOESM4_ESM.docx]

**Fig. S2.** Adjusted Hazard Ratios for All-cause Mortality, Cancer Mortality, and Noncancer Mortality in PHQ-9 ≥5/ Sleep disturbances– Participants Compared with the PHQ-9 <5/ sleep disturbances–, Stratified by Age, Sex, Educational Attainment, Work Status, and Use of Antidepressants

A All-cause mortality

|  | Unweighted patients/total, No. | Unweighted patients/total, No. |  |  |  |
| --- | --- | --- | --- | --- | --- |
| Subgroup | PHQ-9 ≥5/ sleep disturbances– | PHQ-9 <5/ sleep disturbances– | HR (95% CI) |  | *P* value |
| All patients | 57/161 | 379/1623 | 1.87 (1.37–2.55) |  | <0.001 |
| Age, y |  |  |  |  |  |
| 20-69 | 14/81 | 85/760 | 1.39 (0.71–2.69) |  | 0.337 |
| 70 or older | 43/80 | 294/863 | 2.36 (1.64–3.37) |  | <0.001 |
| Sex |  |  |  |  |  |
| Male | 27/55 | 244/891 | 2.00 (1.28–3.13) |  | 0.002 |
| Female | 30/106 | 135/732 | 1.72 (1.10–2.70) |  | 0.018 |
| Educational attainment |  |  |  |  |  |
| Less than some college | 32/81 | 203/660 | 1.92 (1.25–2.94) |  | 0.003 |
| Some college or above | 25/80 | 176/962 | 1.98 (1.23–3.17) |  | 0.005 |
| Work status |  |  |  |  |  |
| Nonemployed | 55/124 | 342/1120 | 1.98 (1.44–2.73) |  | <0.001 |
| Employed | 2/37 | 36/497 | 1.03 (0.21–4.94) |  | 0.972 |
| Use of antidepressants |  |  |  |  |  |
| No | 35/94 | 306/1359 | 2.37 (1.59–3.53) |  | <0.001 |
| Yes | 22/66 | 68/247 | 1.40 (0.84–2.34) |  | 0.202 |
|  |  |  |  |  |  |
|  |  |  |  |  |  |

B Cancer-specific mortality

|  | Unweighted patients/total, No. | Unweighted patients/total, No. |  |  |  |
| --- | --- | --- | --- | --- | --- |
| Subgroup | PHQ-9 ≥5/ sleep disturbances– | PHQ-9 <5/ sleep disturbances– | HR (95% CI) |  | P value |
| All patients | 20/161 | 132/1623 | 1.95 (1.16–3.27) |  | 0.012 |
| Age, y |  |  |  |  |  |
| 20-69 | 5/81 | 49/760 | 0.87 (0.30–2.51) |  | 0.796 |
| 70 or older | 15/80 | 83/863 | 3.60 (1.94–6.68) |  | <0.001 |
| Sex |  |  |  |  |  |
| Male | 9/55 | 93/891 | 1.51 (0.68–3.34) |  | 0.309 |
| Female | 11/106 | 39/732 | 2.06 (0.99–4.28) |  | 0.052 |
| Educational attainment |  |  |  |  |  |
| Less than some college | 9/81 | 81/660 | 1.23 (0.57–2.68) |  | 0.593 |
| Some college or above | 11/80 | 51/962 | 3.22 (1.56–6.67) |  | 0.002 |
| Work status |  |  |  |  |  |
| Nonemployed | 20/124 | 115/1120 | 2.25 (1.33–3.83) |  | 0.003 |
| Employed | 0/37 | 17/497 | Infinite |  | 0.998 |
| Use of antidepressants |  |  |  |  |  |
| No | 11/94 | 110/1359 | 1.85 (0.92–3.73) |  | 0.083 |
| Yes | 9/66 | 20/247 | 2.09 (0.89–4.91) |  | 0.090 |
|  |  |  |  |  |  |
|  |  |  |  |  |  |

C Noncancer mortality

|  | Unweighted patients/total, No. | Unweighted patients/total, No. |  |  |  |
| --- | --- | --- | --- | --- | --- |
| Subgroup | PHQ-9 ≥5/ sleep disturbances– | PHQ-9 <5/ sleep disturbances– | HR (95% CI) |  | P value |
| All patients | 37/161 | 247/1623 | 1.83 (1.24–2.71) |  | 0.002 |
| Age, y |  |  |  |  |  |
| 20-69 | 9/81 | 36/760 | 2.06 (0.85–4.98) |  | 0.107 |
| 70 or older | 28/80 | 211/863 | 1.98 (1.27–3.10) |  | 0.003 |
| Sex |  |  |  |  |  |
| Male | 18/55 | 151/891 | 2.19 (1.27–3.78) |  | 0.005 |
| Female | 19/106 | 96/732 | 1.53 (0.85–2.74) |  | 0.153 |
| Educational attainment |  |  |  |  |  |
| Less than some college | 23/81 | 122/660 | 2.41 (1.44–4.04) |  | <0.001 |
| Some college or above | 14/80 | 125/962 | 1.52 (0.81–2.84) |  | 0.192 |
| Work status |  |  |  |  |  |
| Nonemployed | 35/124 | 227/1120 | 1.87 (1.24–2.80) |  | 0.003 |
| Employed | 2/37 | 19/497 | 2.70 (0.52–13.92) |  | 0.235 |
| Use of antidepressants |  |  |  |  |  |
| No | 24/94 | 196/1359 | 2.72 (1.66–4.44) |  | <0.001 |
| Yes | 13/66 | 48/247 | 1.19 (0.61–2.30) |  | 0.611 |
|  |  |  |  |  |  |
|  |  |  |  |  |  |

Abbreviations: PHQ-9, Patient Health Questionnaire-9; HR, hazard ratio.

Each stratification was adjusted for age, sex (male/female), race and ethnicity (Mexican American, other Hispanic, non-Hispanic White, non-Hispanic Black, other race or ethnicity [including American Indian/Alaska Native/Pacific Islander, Asian, multiracial]), educational attainment (<high school graduate, high school graduate or general equivalency diploma, ≥Some college), marital status (married, never married, living with partner, other [including widowed, divorced, separated individuals]), family poverty income ratio (≤1.3, 1.3–3.5, ＞3.5), work status (nonemployed, part time [1–34 h/wk], full time [≥35 h/wk]), National Health and Nutrition Examination Survey cycles (2007–2008, 2009–2010, 2011–2012, 2013–2014, 2015–2016, 2017–2018), diabetes (yes/no), hypertension (yes/no), hypercholesterolemia (yes/no), the number of cancer types (1, 2, ≥3), the number of years since the first cancer diagnosis, use of antidepressants (yes/no), and sleep duration except the stratification factor itself. Squares indicate hazard ratios (HRs), with horizontal lines indicating 95% CIs.
