# Supplementary material for: Association of depressive symptoms and sleep disturbances with survival among US adult cancer survivors
Source: BMC Med. 2024 Jun 5;22:225. doi: 10.1186/s12916-024-03451-7 (PMC11151538; doi:10.1186/s12916-024-03451-7)
Supplement: Supplementary file 5 — Additional file 5: Table S3. Sensitivity Analyses for All-cause Mortality According to PHQ-9 Score and Sleep Disturbances. [file 12916_2024_3451_MOESM5_ESM.docx]

**Table S3.** Sensitivity Analyses for All-cause Mortality According to PHQ-9 Score and Sleep Disturbances

| **Analysis** | **Sleep disturbances** | **Death/No.** | **Hazard ratio (95% CI)^a^** | ***P* value** |
| --- | --- | --- | --- | --- |
| **Exclusion of deaths during the first two years of follow-up** | | | | |
| PHQ-9 score 0–4 | No | 310/1554 | 1 [Reference] | Reference |
|  | Yes | 91/510 | 1.03 (0.80–1.33) | 0.805 |
| PHQ-9 score 5–9 | No | 29/112 | 1.45 (0.94–2.21) | 0.091 |
|  | Yes | 55/324 | 0.93 (0.68–1.28) | 0.665 |
| PHQ-9 score ≥10 | No | 10/31 | 2.89 (1.43–5.85) | 0.003 |
|  | Yes | 35/259 | 0.99 (0.66–1.48) | 0.966 |
| **Exclusion of non-Hispanic Black participants** | | | | |
| PHQ-9 score 0–4 | No | 319/1374 | 1 [Reference] | Reference |
|  | Yes | 97/462 | 1.06 (0.83–1.36) | 0.629 |
| PHQ-9 score 5–9 | No | 39/110 | 1.66 (1.14–2.41) | 0.008 |
|  | Yes | 70/298 | 1.10 (0.83–1.46) | 0.521 |
| PHQ-9 score ≥10 | No | 12/29 | 3.01 (1.59–5.71) | <0.001 |
|  | Yes | 43/239 | 1.15 (0.80–1.65) | 0.464 |

Abbreviations: PHQ-9, Patient Health Questionnaire-9.

^a^ Adjusted for age, sex (male/female), race and ethnicity (Mexican American, other Hispanic, non-Hispanic White, non-Hispanic Black, other race or ethnicity [including American Indian/Alaska Native/Pacific Islander, Asian, multiracial]), educational attainment (<high school graduate, high school graduate or general equivalency diploma, ≥Some college), marital status (married, never married, living with partner, other [including widowed, divorced, separated individuals]), family poverty income ratio (≤1.3, 1.3–3.5, ＞3.5), work status (nonemployed, part time [1–34 h/wk], full time [≥35 h/wk]), National Health and Nutrition Examination Survey cycles (2007–2008, 2009–2010, 2011–2012, 2013–2014, 2015–2016, 2017–2018), diabetes (yes/no), hypertension (yes/no), hypercholesterolemia (yes/no), the number of cancer types (1, 2, ≥3), the number of years since the first cancer diagnosis, use of antidepressants (yes/no), and sleep duration.
