# Supplementary material for: Association of depressive symptoms and sleep disturbances with survival among US adult cancer survivors
Source: BMC Med. 2024 Jun 5;22:225. doi: 10.1186/s12916-024-03451-7 (PMC11151538; doi:10.1186/s12916-024-03451-7)
Supplement: Supplementary file 6 — Additional file 6: Table S4. Sensitivity Analyses for Cancer-specific Mortality According to PHQ-9 Score and Sleep Disturbances. [file 12916_2024_3451_MOESM6_ESM.docx]

**Table S4.** Sensitivity Analyses for Cancer-specific Mortality According to PHQ-9 Score and Sleep Disturbances

| **Analysis** | **Sleep disturbances** | **Death/No.** | **Hazard ratio (95% CI)^a^** | ***P* value** |
| --- | --- | --- | --- | --- |
| **Exclusion of deaths during the first two years of follow-up** | | | | |
| PHQ-9 score 0–4 | No | 94/1554 | 1 [Reference] | Reference |
|  | Yes | 28/510 | 0.95 (0.61–1.50) | 0.833 |
| PHQ-9 score ≥5 | No | 12/143 | 1.62 (0.84–3.15) | 0.153 |
|  | Yes | 29/583 | 0.84 (0.52–1.37) | 0.485 |
| **Exclusion of non-Hispanic Black participants** | | | | |
| PHQ-9 score 0–4 | No | 111/1374 | 1 [Reference] | Reference |
|  | Yes | 26/462 | 0.77 (0.49–1.21) | 0.254 |
| PHQ-9 score ≥5 | No | 16/139 | 1.77 (0.99–3.17) | 0.054 |
|  | Yes | 43/537 | 1.14 (0.76–1.70) | 0.536 |

Abbreviations: PHQ-9, Patient Health Questionnaire-9.

^a^ Adjusted for age, sex (male/female), race and ethnicity (Mexican American, other Hispanic, non-Hispanic White, non-Hispanic Black, other race or ethnicity [including American Indian/Alaska Native/Pacific Islander, Asian, multiracial]), educational attainment (<high school graduate, high school graduate or general equivalency diploma, ≥Some college), marital status (married, never married, living with partner, other [including widowed, divorced, separated individuals]), family poverty income ratio (≤1.3, 1.3–3.5, ＞3.5), work status (nonemployed, part time [1–34 h/wk], full time [≥35 h/wk]), National Health and Nutrition Examination Survey cycles (2007–2008, 2009–2010, 2011–2012, 2013–2014, 2015–2016, 2017–2018), diabetes (yes/no), hypertension (yes/no), hypercholesterolemia (yes/no), the number of cancer types (1, 2, ≥3), the number of years since the first cancer diagnosis, use of antidepressants (yes/no), and sleep duration.
