# Supplementary material for: Association of depressive symptoms and sleep disturbances with survival among US adult cancer survivors
Source: BMC Med. 2024 Jun 5;22:225. doi: 10.1186/s12916-024-03451-7 (PMC11151538; doi:10.1186/s12916-024-03451-7)
Supplement: Supplementary file 7 — Additional file 7: Table S5. Sensitivity Analyses for Noncancer Mortality According to PHQ-9 Score and Sleep Disturbances. [file 12916_2024_3451_MOESM7_ESM.docx]

**Table S5.** Sensitivity Analyses for Noncancer Mortality According to PHQ-9 Score and Sleep Disturbances

| **Analysis** | **Sleep disturbances** | **Death/No.** | **Hazard ratio (95% CI)^a^** | ***P* value** |
| --- | --- | --- | --- | --- |
| **Exclusion of deaths during the first two years of follow-up** | | | | |
| PHQ-9 score 0–4 | No | 216/1554 | 1 [Reference] | Reference |
|  | Yes | 63/510 | 1.08 (0.79–1.47) | 0.630 |
| PHQ-9 score 5–9 | No | 20/112 | 1.43 (0.85–2.40) | 0.179 |
|  | Yes | 39/324 | 1.00 (0.68–1.46) | 0.991 |
| PHQ-9 score ≥10 | No | 7/31 | 3.49 (1.49–8.22) | 0.004 |
|  | Yes | 22/259 | 1.08 (0.66–1.76) | 0.771 |
| **Exclusion of non-Hispanic Black participants** | | | | |
| PHQ-9 score 0–4 | No | 208/1374 | 1 [Reference] | Reference |
|  | Yes | 71/462 | 1.27 (0.94–1.70) | 0.119 |
| PHQ-9 score 5–9 | No | 27/110 | 1.71 (1.09–2.68) | 0.020 |
|  | Yes | 41/298 | 0.99 (0.68–1.44) | 0.954 |
| PHQ-9 score ≥10 | No | 8/29 | 3.77 (1.69–8.40) | 0.001 |
|  | Yes | 29/239 | 1.37 (0.88–2.13) | 0.169 |

Abbreviations: PHQ-9, Patient Health Questionnaire-9.

^a^ Adjusted for age, sex (male/female), race and ethnicity (Mexican American, other Hispanic, non-Hispanic White, non-Hispanic Black, other race or ethnicity [including American Indian/Alaska Native/Pacific Islander, Asian, multiracial]), educational attainment (<high school graduate, high school graduate or general equivalency diploma, ≥Some college), marital status (married, never married, living with partner, other [including widowed, divorced, separated individuals]), family poverty income ratio (≤1.3, 1.3–3.5, ＞3.5), work status (nonemployed, part time [1–34 h/wk], full time [≥35 h/wk]), National Health and Nutrition Examination Survey cycles (2007–2008, 2009–2010, 2011–2012, 2013–2014, 2015–2016, 2017–2018), diabetes (yes/no), hypertension (yes/no), hypercholesterolemia (yes/no), the number of cancer types (1, 2, ≥3), the number of years since the first cancer diagnosis, use of antidepressants (yes/no), and sleep duration.
