# Supplementary material for: Association of depressive symptoms and sleep disturbances with survival among US adult cancer survivors
Source: BMC Med. 2024 Jun 5;22:225. doi: 10.1186/s12916-024-03451-7 (PMC11151538; doi:10.1186/s12916-024-03451-7)
Supplement: Supplementary file 8 — Additional file 8: Table S6. Joint Association of PHQ-9 Score and Sleep Disturbances with All-Cause, Cancer, and Noncancer Mortality Among US Cancer Survivors Aged 20 Years or Older, National Health and Nutrition Examination Survey 2007–2018: Multiple Imputation for Sensitivity Analysis. [file 12916_2024_3451_MOESM8_ESM.docx]

**Table S6.** Joint Association of PHQ-9 Score and Sleep Disturbances with All-Cause, Cancer, and Noncancer Mortality Among US Cancer Survivors Aged 20 Years or Older, National Health and Nutrition Examination Survey 2007–2018: Multiple Imputation for Sensitivity Analysis

|  | | **Hazard ratio (95% CI)** | |
| --- | --- | --- | --- |
| **Mortality outcome** | **Sleep disturbances** | **MV model 1^a^** | **MV model 2^a, b^** |
| **All causes** |  |  |  |
| PHQ-9 score 0–4 | No | 1 [Reference] | 1 [Reference] |
|  | Yes | 0.98 (0.79–1.21) | 1.02 (0.82–1.26) |
| PHQ-9 score 5–9 | No | 1.71 (1.24–2.35) | 1.58 (1.15–2.18) |
|  | Yes | 1.15 (0.90–1.47) | 1.12 (0.87–1.43) |
| PHQ-9 score ≥10 | No | 2.61 (1.47–4.62) | 2.47 (1.39–4.38) |
|  | Yes | 1.27 (0.95–1.71) | 1.16 (0.86–1.58) |
| **Cancer** |  |  |  |
| PHQ-9 score 0–4 | No | 1 [Reference] | 1 [Reference] |
|  | Yes | 0.88 (0.61–1.28) | 0.94 (0.64–1.36) |
| PHQ-9 score ≥5 | No | 1.81 (1.11–2.93) | 1.80 (1.10–2.94) |
|  | Yes | 1.10 (0.78–1.55) | 1.14 (0.80–1.63) |
| **Noncancer** |  |  |  |
| PHQ-9 score 0–4 | No | 1 [Reference] | 1 [Reference] |
|  | Yes | 1.04 (0.80–1.35) | 1.07 (0.82–1.39) |
| PHQ-9 score 5–9 | No | 1.71 (1.16–2.54) | 1.52 (1.02–2.26) |
|  | Yes | 1.13 (0.83–1.54) | 1.06 (0.77–1.45) |
| PHQ-9 score ≥10 | No | 3.10 (1.50–6.40) | 2.77 (1.34–5.74) |
|  | Yes | 1.52 (1.06–2.19) | 1.31 (0.90–1.91) |

Abbreviations: PHQ-9, Patient Health Questionnaire-9; MV, multivariable.

^a^ Multivariable model adjusted for age, sex (male/female), race and ethnicity (Mexican American, other Hispanic, non-Hispanic White, non-Hispanic Black, other race or ethnicity [including American Indian/Alaska Native/Pacific Islander, Asian, multiracial]), educational attainment (<high school graduate, high school graduate or general equivalency diploma, ≥Some college), marital status (married, never married, living with partner, other [including widowed, divorced, separated individuals]), family poverty income ratio (≤1.3, 1.3–3.5, ＞3.5), work status (nonemployed, part time [1–34 h/wk], full time [≥35 h/wk]), and National Health and Nutrition Examination Survey cycles (2007–2008, 2009–2010, 2011–2012, 2013–2014, 2015–2016, 2017–2018).

^b^ Additionally adjusted for diabetes (yes/no), hypertension (yes/no), hypercholesterolemia (yes/no), the number of cancer types (1, 2, ≥3), the number of years since the first cancer diagnosis, use of antidepressants (yes/no), and sleep duration.
